# Independent determinants of disease-related quality of life in COPD – scope for nonpharmacologic interventions?

**DOI:** 10.2147/COPD.S152955

**Published:** 2018-01-09

**Authors:** Sarah B Brien, Beth Stuart, Andrew P Dickens, Tony Kendrick, Rachel E Jordan, Paymane Adab, Mike Thomas

**Affiliations:** 1 Primary Care and Population Sciences, Faculty of Medicine, University of Southampton, Southampton, Hampshire; 2 Institute of Applied Health Research, College of Medical and Dental Sciences, University of Birmingham, Birmingham, Warwickshire, UK

**Keywords:** chronic obstructive pulmonary disease, quality of life, health status, survey, psychological, dysfunctional breathing, breathlessness, illness perception, depression

## Abstract

**Purpose:**

Quality-of-life (QoL) scores in chronic obstructive pulmonary disease (COPD) have a weak relationship with physiologic impairment. We investigated factors associated with poor QoL, focusing on psychological measures potentially amenable to intervention.

**Patients and methods:**

We utilized a pre-existing Birmingham (UK) COPD cohort to assess factors associated with QoL impairment (COPD Assessment Test [CAT] scores). Univariate and multivariate regression models were constructed from three categories of variables: demographic, lung function/COPD-related symptoms, and psychosocial/behavioral factors.

**Results:**

Analyses were based on self-report questionnaire data from 735 participants. The multivariate model of variables independently associated with CAT included depression, dysfunctional breathing symptoms (Nijmegen score), and illness perception, in addition to COPD symptoms (wheeze, cough), exercise capacity, breathlessness, exacerbations, and deprivation; this model explained 72% of CAT score variation. In a dominance analysis assessing the relative contribution of variables, similar contributions were made by breathlessness (20.2%), illness perception (19.8%), dysfunctional breathing symptoms (17.5%), and depression (12.5%) with other variables contributing <5%.

**Conclusion:**

Psychological factors significantly contribute to disease-specific QoL impairment in COPD, and potentially explain the mismatch between objective physiologic impairment and patients’ experience of their disease. Interventions targeting psychological factors, illness perception, and dysfunctional breathing should be assessed.

## Introduction

Chronic obstructive pulmonary disease (COPD) is a major cause of morbidity and mortality and a global public health problem.^1^ As a complex, multifaceted disease, it affects patients in many ways and results in significant quality-of-life (QoL) impairment.^2^ However, QoL varies greatly between individuals, and is only weakly associated with physiologic factors such as percentage predicted forced expiratory volume in 1 second (FEV_1_).^3^ Anxiety and depression are common, although frequently unrecognized and untreated,^4^ and are associated with poor COPD outcomes.^5^–^7^ Previous research suggests that a variety of disease-related and patient-related factors may be associated with QoL impairment in COPD.^3^ In addition to biologic factors (such as lung function and inflammation) and demographic/socioeconomic factors, there is evidence that QoL is affected by comorbidities, particularly psychological conditions,^8^–^11^ and by psychological constructs such as illness perception.^12^ The relative importance of these different factors is currently unclear.

Understanding the determinants of QoL impairment in COPD may highlight modifiable factors that could be targeted to minimize disease impact and to help patients to cope better with the consequences of having an incurable long-term condition. In particular, psychological and behavioral interventions may be appropriate for selected patients. Nonpharmacologic interventions are acceptable to many patients, particularly those with significantly impaired QoL and those with “disproportionate” QoL impairment in relation to their lung function impairment.^13^ The importance of a “personalized medicine” strategy for managing airways disease has recently been emphasized, aiming to target appropriate treatments, both pharmacologic and nonpharmacologic, on potentially modifiable factors in well-characterized individual patients.^14^ We hypothesized that a range of psychological, social, perceptual, and behavioral factors, measured using validated patient-reported outcome measures, would be independently associated with QoL in patients with COPD.

## Methods

### Study design

We report a cross-sectional analysis of data from the Birmingham COPD cohort study.^15^ The current analysis investigated factors independently influencing disease-specific QoL (assessed by the COPD Assessment Test [CAT])^16^ and generic health-related (HR) QoL score using the 5 level EuroQoL questionnaire (EQ-5D 5L),^17^ and estimated the magnitude of contribution of different contributory factors, with a particular focus on clarifying the relative contribution of psychosocial and behavioral factors.

### Subjects and setting

The Birmingham COPD cohort study is described fully elsewhere,^18^ but briefly consists of three patient groups recruited from 71 primary care practices in the West Midlands, UK during the period May 2012–June 2014: 1) patients with diagnosed COPD on general practitioner registers; 2) newly identified COPD patients from a linked case-finding study;^15^ and 3) participants from the case-finding study with chronic respiratory symptoms but without airflow obstruction. Cohort participants were characterized at baseline with a series of questionnaires and objective measurements (including the Medical Research Council [MRC] breathlessness scale,^19^ assessments of lung function, muscle strength, and exercise capacity as sit/stand repetitions with Borg breathlessness scores^20^ pre- and post-exercise). Cohort participants were invited to complete review questionnaires at 6-monthly intervals for 3 years including items regarding health, lifestyle, health-related quality of life (HRQoL), exacerbations, health care usage, and medical conditions; health resource use information was also collected. At the time of this analysis, the full cohort consisted of 2,188 patients. Ethical approval was provided by the National Research Ethics Service Committee, West Midlands, Solihull, UK (ref: 11/WM/0304). All participants provided written informed consent for this study.

For the purposes of the current analysis, an additional questionnaire set was administered at one time point per patient, assessing symptoms of dysfunctional breathing (Nijmegen questionnaire),^21^ depression (PHQ-9),^22^ anxiety (GAD-7),^23^ illness perceptions (Brief Illness Perception Questionnaire, IPQ),^24^ and agoraphobic avoidance.^25^ Questionnaires were posted to participants with an explanatory letter and a return envelope, with one reminder sent 2 weeks after the initial mailing.

Patients were included in our analysis if they 1) had an existing COPD diagnosis or were identified from the case-finding trial and 2) met the spirometric criteria for COPD, based on UK guideline definitions (FEV_1_/FVC [forced vital capacity] <0.7) at the baseline assessment.

### Statistical methods

Descriptive statistics are reported on all variables. Univariate associations between disease-specific QoL (CAT score) and other variables were analyzed using linear regression. Initially we fitted univariate regression models to determine which variables were significantly associated with CAT at the 5% level. These variables were taken forward into multivariate regression models to determine which were independently predictive in mutually adjusted analyses. We considered variables within three categories: 1) demographic characteristics (age, sex, body mass index [BMI], socioeconomic status [Index of Multiple Deprivation {IMD}, as quintiles],^26^ employment status, comorbidity); 2) COPD-related factors (FEV_1_% predicted, physical activity, chronic cough/phlegm, chronic wheeze, breathlessness, exacerbations, hospitalizations); and 3) psychosocial and behavioral factors (anxiety, depression, dysfunctional breathing symptoms, illness perception, agoraphobic avoidance).

Those variables that were independently predictive of CAT score at the 5% significance level within each of these categories were taken forward in a final regression model to determine which variables were independently predictive overall, and to explore whether psychological measures had an association with CAT score independent of patient characteristics and COPD-related symptoms. In the multivariate models, only cases without missing data in any field were included and none of the missing data were imputed. Although this does result in a reduction in the sample size, as the purpose of this study was to explore the relationship between variables and to determine which were most highly predictive of HRQoL, it was important that participants contributed data on all variables. A multiple imputation model would have required some assumption of the likely distribution of missing values and might have introduced bias into our exploratory analysis by imposing a likely relationship between variables and between the variables and HRQoL.

The R-squared value was used in order to quantify the extent to which the variables explained the variation in CAT score. To determine the contribution that each predictor made to the overall variance, and therefore their relative importance, a dominance analysis was undertaken.^27^ Dominance analysis examines the change in R-squared from adding a variable to all possible subset regression models and then averaging across all possible models. In this way, it is possible to obtain a general dominance weight and thereby partition the R-squared value among the predictors. All variables that were significant in their respective categories were included in the dominance analysis.

A further analysis was conducted using the same methodology with generic HR QoL score EQ-5D 5L rather than the disease-specific CAT score as the outcome variable.

Analyses were carried out in Stata v14 (StataCorp. 2015. Stata Statistical Software: Release 14. College Station, TX: StataCorp LP).

## Results

### Eligible population

One thousand six hundred three cohort participants meeting entry criteria and with valid spirometry readings were posted the additional questionnaire sets, and 1,233 were returned (76.9%). Of these, 181 were excluded from analysis as they did not have obstructive spirometry (FEV_1_/FVC ratio <0.70), and a further 317 as they did not have a valid CAT score, leaving an analysis sample of 735 participants (consisting of 599 previously diagnosed COPD subjects and 136 identified through case finding). The case-found patients had milder disease, with better-preserved lung function, less breathlessness, chronic cough and wheeze, and a history of fewer exacerbations (Table 1).

A large variation in CAT score was observed (mean, SD CAT score 17.9, 8.3). Case-found participants reported less COPD impact compared to those with diagnosed COPD (14.0, 7.6 vs 18.7, 8.2, respectively).

### Univariate and multivariate associations with COPD-related QoL

#### Demographic characteristics

Four patient characteristics were significantly associated with higher CAT score: higher level of deprivation (IMD status), being currently unemployed, younger age, and being a current smoker (Table 2). Although these associations were statistically significant at the 5% level, the R-squared values were low. The highest R-squared value was for deprivation and even that explained only 5.7% of the variation in CAT scores.

#### Lung function and COPD-related symptoms

The independent predictors in this category were chronic wheeze, chronic cough/phlegm, exercise capacity at baseline (sit/stand repetitions), MRC score, Borg breathlessness pre- and post-exercise scores, and having ≥2 exacerbations in the previous 12 months (Table 3). The MRC score was the strongest predictor, explaining 46% of the variation in CAT score in this category of factors. The other variables explained no more than 16% each, with lung function impairment (% predicted FEV_1_) independently explaining under 6% of CAT variation in this category group.

#### Psychosocial and behavioral factors

The PHQ-9 (measuring depression), Nijmegen questionnaire (measuring symptoms of dysfunctional breathing), and the brief IPQ (measuring illness perceptions) were all significantly associated with CAT in this group of factors, and explained 34%, 38%, and 45% of the variance in CAT, respectively. Anxiety (GAD-7) scores were not significantly independently associated and agoraphobic avoidance scores were only weakly associated (Table 4).

#### Full multivariate model and dominance analysis

A full multivariate model was constructed including all variables independently associated with CAT from each category (Table 5). This model only included people without missing data for any variable (n=476). In this model, depression (PHQ9), dysfunctional breathing symptoms (Nijmegen score), illness perception (IPQ), symptoms of both chronic wheeze and chronic phlegm/cough, exercise capacity (sit/stand repetitions), breathlessness (MRC score and Borg score post-exercise), having ≥2 exacerbations in the previous year, and deprivation (IMD quintile) were all significantly associated with CAT at the 5% level. This model explains 72.2% of the observed variation in CAT.

In the dominance analysis, the largest contributions to variation in CAT scores were made by functional breathlessness (MRC score, 20.2% of R-squared value), with illness perception (IPQ) providing only a slightly lower contribution (19.8%). Dysfunctional breathing symptoms (Nijmegen questionnaire, 17.5%) and depression (PHQ9, 12.5%) were the next most important contributors to the CAT variation, and other variables contributed 5% or less.

### Analysis using generic HRQoL (EQ-5D 5L)

A further analysis was performed with generic QoL score (EQ-5D 5L) as the outcome measure (Table 6). Generally, the same variables emerged as important predictors, although measures of depression (PHQ-9) and anxiety (GAD-7) were more strongly associated with EQ-5D 5L than with CAT scores. This model predicted 60.4% of the variation in the EQ-5D 5L, with the largest contributions made by depression (PHQ-9, 23.1%), functional breathlessness (MRC score, 20.1%), illness perception (IPQ, 16.0%), anxiety (GAD-7, 14.0%), and symptoms of dysfunctional breathing (Nijmegen score, 13.3%). The other significant variables (exercise capacity, BMI, physical activity levels, breathlessness pre-exercise, smoking status, deprivation, chronic wheeze, and employment status) all made smaller contributions (5% or less).

## Discussion

The stimulus for this study was the observation that there is a large discrepancy at an individual level between the objective, biologic severity of COPD and the impact of the disease on the patient, as assessed by functional impairment and effects on QoL. We aimed to assess and quantify the relative contributions of psychosocial and behavioral factors on QoL, in order to identify candidate targets for future interventions. In this cohort of community-based patients predominantly having physiologically mild-to-moderate COPD, we observed a wide variation in disease-related QoL scores, with a mean (SD) CAT score of 17.9 (8.3). The reference values for CAT scores suggest that a score of <10 indicates low impact, 10–20 moderate impact, and >20 high impact,^28^ showing that the patients in our cohort spanned a wide range of perceived QoL impairment from their COPD, ranging from low to very high impact. As in previous research, a weak relationship was observed between physiologically assessed lung function and QoL.^29^

We found, unsurprisingly, that a major independent contribution to impaired QoL came from functional, activity-related breathlessness, with the MRC breathlessness score explaining 20% of the variation in both disease-specific (CAT) and generic QoL scores (EQ-5D 5L). However, we also found that both pessimistic health beliefs (as measured by the validated IPQ) and depressed psychological state (assessed by the PHQ-9) have major independent impacts on patients’ experience of their COPD, explaining 20% and 12%, respectively, of the variation in CAT scores and high proportions (16% and 23%, respectively) of the variation in EQ-5D 5L. In addition, we found that dysfunctional breathing (measured by the Nijmegen Questionnaire) independently explained a large proportion of QoL variability (17% of CAT and 13% of EQ-5D 5L). All these factors are potentially amenable to intervention. Overall, the models we constructed explained over 70% of the variation in disease-specific QoL and over 60% of that in generic QoL.

Our findings are in keeping with other smaller studies suggesting that psychological factors and illness perceptions are important determinants of well-being, overshadowing the influence of “harder” factors such as lung function in COPD.^3^,^8^,^12^,^30^,^31^ Illness perception is a construct of the cognitive representations and beliefs that patients have about their illness, and has been found to be an important determinant of behavior and to be associated with a number of important outcomes, including treatment adherence and functional recovery.^32^ The Brief IPQ is a widely used and validated tool with good psychometric properties.^33^ There is limited research linking poor illness perception with disability and impaired QoL in COPD,^34^ and no interventional studies that we are aware of. There is emerging evidence that brief, straightforward psychoeducational interventions can help to modify negative illness beliefs and lead to improvements over a range of different health outcomes.^35^ In view of the findings we report in this study, there is a case for developing interventional studies based on strategies to improve illness perception in COPD.

There is consistent evidence that depression and anxiety are common comorbidities in patients with COPD and are associated with poor outcomes.^3^–^10^,^36^,^37^ There is, however, surprisingly little evidence to support the use of psychological interventions in managing COPD, or to clarify which interventions are most effective and acceptable.^38^ Although there is some preliminary evidence to support interventions such as relaxation, cognitive behavioral therapy, self-management, and antidepressant medication in COPD, the data are limited and mainly consisted of small studies.^38^ Qualitative evidence suggests that psychological interventions are acceptable to COPD patients with disproportionately impaired QoL in relation to lung function impairment, particularly interventions based on nonpharmacologic strategies.^13^ Our finding that a large proportion of QoL variation is explained by psychological factors supports the need for interventional studies.

A novel contribution of this study was to include a measure of dysfunctional breathing, the Nijmegen Questionnaire, which was found to be a major contributor to the variation in both generic and disease-specific QoL. Although this questionnaire has not been validated for use in COPD, it has been widely used in other airways diseases including asthma,^39^ with breathing retraining interventions shown to improve QoL scores.^40^ The significant independent association of Nijmegen Questionnaire score to QoL improvement in patients with COPD indicates that breathing retraining may also be a possible strategy to improve QoL in people with COPD.

A strength of this study was the availability of data from a large well-characterized UK COPD cohort, containing a relatively representative sample of patients with mild and moderate COPD treated in primary care. The large sample size, compared with much smaller previous studies (two studies had samples of <100),^12^,^30^ and the collection of data on a wide panel of disease-related factors allowed us to examine the relative contribution of different factors relating to QoL impairment in COPD. We were able to include a wide range of potential explanatory variables in the models, which were more inclusive than those in previous studies. Also, unlike previous studies that used only generic QoL tools,^31^ we used both generic and disease-specific instruments. Similar messages emerged from our study for disease-specific and generic QoL instruments.

A limitation of our study is that it is cross-sectional, describing associations between measured factors and QoL that cannot be assumed as causal, and there is no certainty that interventions targeting these factors will result in clinically important improvements in QoL. It does, however, provide a justification for future interventional studies targeted on improving illness perception, improving depression and correcting dysfunctional breathing in COPD patients, particularly in those with disproportionately impaired QoL in relation to lung function impairment.

## Conclusion

COPD is a distressing and progressive condition that makes life miserable for many, and a holistic and multidimensional, personalized approach is needed.^14^ The clinical focus in COPD is generally directed toward biologic factors such as physiologic impairment and airways inflammation, with a predominant focus on pharmacologic interventions. However, along with smoking cessation, nonpharmacologic approaches are also very important in improving patient outcomes, with pulmonary rehabilitation now recognized as being a key element in overall disease management. This study shows that there are a number of factors associated with QoL impairment that could potentially be modified through suitable nonpharmacologic interventions focused on appropriately characterized patients, and supports the need for future interventional studies.

## Figures and Tables

**Table 1:** Baseline characteristics

Characteristics	Previously diagnosed (n=599)	Case found (n=136)	Total (n=735)
Mean CAT score (SD)	18.7 (8.2)	14.0 (7.6)	17.9 (8.3)
CAT score <10 (%)	133 (22.2)	63 (46.3)	196 (26.7)
CAT score 10–20 (%)	254 (42.40)	58 (42.7)	312 (42.5)
CAT score >20 (%)	212 (35.4)	15 (11.0)	227 (30.9)
Predicted FEV_1_ (%)	66.1 (20.8)	84.7 (15.6)	69.6 (21.2)
Severe COPD^a^ (%)	137/599 (22.9)	1/136 (0.7)	138/735 (18.9)
Male (%)	401/599 (66.9)	83/136 (61.0)	484/735 (65.9)
Age	68.9 (8.4)	65.4 (8.6)	68.3 (8.5)
BMI (%)			
Normal	144/586 (24.6)	26/121 (21.5)	170/707 (24.1)
Underweight	11/586 (1.9)	2/121 (1.7)	13/707 (1.8)
Overweight	251/586 (42.8)	47/121 (38.8)	298/707 (42.2)
Obese	180/586 (30.7)	46/121 (38.0)	226/707 (32.0)
Current working status (%)			
Employed	62/590 (10.5)	33/135 (24.4)	95/725 (13.1)
Unemployed	167/590 (28.3)	28/135 (20.7)	195/725 (26.9)
Retired	361/590 (61.2)	74/135 (54.8)	435/725 (60.0)
IMD score quintile (%)			
1 (most deprived)	129/599 (21.5)	31/136 (22.8)	160/735 (21.8)
2	155/599 (25.9)	33/136 (24.3)	188/735 (25.6)
3	95/599 (15.9)	34/136 (25.0)	129/735 (17.6)
4	126/599 (21.0)	20/136 (14.7)	146/735 (19.9)
5 (least deprived)	94/599 (15.7)	18/136 (13.2)	112/735 (15.2)
Physical activity (IPAQ) score (%)			
Low	189/476 (39.7)	35/120 (29.2)	224/596 (37.6)
Moderate	181/476 (38.0)	42/120 (35.0)	223/596 (37.4)
High	106/476 (22.3)	43/120 (35.8)	149/596 (25.0)
Cough/phlegm for >3 consecutive months/year	384/596 (64.4)	75/135 (55.6)	459/731 (62.8)
Wheeze for consecutive months/year (%)	450/596 (75.5)	83/135 (61.5)	533/731 (72.9)
Exacerbations in the past 12 months^b^ (%)			
None	215/583 (36.9)	89/129 (69.0)	304/712 (42.7)
One	121/583 (20.8)	22/129 (17.1)	143/712 (20.1)
Two or more	247/583 (42.4)	18/129 (14.0)	265/712 (37.2)
Respiratory hospitalization in the last 6 months (%)	57/585 (9.7)	5/129 (3.9)	62/714 (8.7)
Smoking (%)			
Current smoker	144/571 (25.2)	50/129 (38.7)	194/700 (27.7)
Ex-smoker	373/571 (65.3)	64/129 (49.6)	437/700 (62.4)
Never smoked	54/571 (9.5)	15/129 (11.6)	69/700 (9.9)
Comorbidity^c^ (%)	88/549 (16.0)	21/127 (16.5)	109/676 (16.1)
Exercise capacity (sit-to-stand repetition)	18.4 (5.9)	21.4 (6.8)	19.0 (6.1)
Muscle strength (grip strength)	29.7 (10.2)	31.7 (11.5)	30.0 (10.5)
MRC score (%)			
1	110/579 (19.0)	52/133 (39.1)	162/712 (22.8)
2	148/579 (25.6)	40/133 (30.1)	188/712 (26.4)
3	144/579 (24.9)	31/133 (23.3)	175/712 (24.6)
4	81/579 (14.0)	6/133 (4.5)	87/712 (12.2)
5	96/579 (16.6)	4/133 (3.0)	100/712 (14.0)

**Notes:**

a FEV_1_ <50% predicted.

b Exacerbations defined based on reported prescriptions for antibiotics or steroids for the participant’s lung condition.

c Comorbidity defined as reported diagnosis of one or more of cancer, diabetes, cardiovascular disease, osteoporosis, fracture, and depression.

**Abbreviations:** BMI, body mass index; CAT, COPD Assessment Test; COPD, chronic obstructive pulmonary disease; FEV_1_, forced expiratory volume in 1 second; IMD, Index of Multiple Deprivation; IPAQ, International Physical Activity Questionnaire; MRC, Medical Research Council.

**Table 2:** Patient demographic characteristics associated with CAT score

Characteristics	Univariate difference in CAT vs reference group (95% CI)	*p*-value	Proportion of CAT explained (R-squared value)	Multivariate difference in CAT vs reference group (95% CI)	*p*-value
Male	−1.13 (−2.52, 0.25)	0.108	0.004		
Age	−0.10 (−0.18, −0.03)	0.009	0.009	−0.002 (−0.10, 0.10)	0.969
BMI					
Normal	Reference		0.014	Reference	
Underweight	2.48 (−2.61, 7.57)	0.340		0.19 (−4.67, 5.06)	0.938
Overweight	−0.27 (−1.97, 1.43)	0.756		0.26 (−1.49, 2.01)	0.770
Obese	2.08 (0.29, 3.88)	0.023		1.73 (−0.10, 3.58)	0.064
Comorbid condition	−0.13 (−2.00, 1.73)	0.889	0.000	−0.01 (−1.83, 1.82)	0.993
Current working status					
Employed	Reference		0.026	Reference	
Unemployed	7.11 (4.96, 9.26)	<0.001		6.21 (3.89, 8.53)	<0.001
Retired	1.83 (−0.10, 3.78)	0.064		2.12 (−0.13, 4.50)	0.064
IMD score quintile					
1 (most deprived)	Reference		0.057		
2	−2.82 (−4.70, −0.96)	0.003		−1.69 (−3.73, 0.34)	0.103
3	−4.89 (−6.94, −2.83)	<0.001		−3.71 (−5.90, −1.51)	0.001
4	−5.14 (−7.12, −3.15)	<0.001		−4.02 (−6.15, −1.88)	<0.001
5 (least deprived)	−6.09 (−8.22, −3.94)	<0.001		−4.31 (−6.61, −2.01)	<0.001
Smoking					
Current smoker	Reference		0.026	Reference	
Ex-smoker	−2.45 (−3.96, −0.94)	0.001		−1.60 (−3.28, 0.06)	0.059
Never smoked	−5.05 (−7.50, −2.59)	<0.001		−4.25 (−6.83, −1.68)	0.001

**Abbreviations:** BMI, body mass index; CAT, COPD Assessment Test; IMD, Index of Multiple Deprivation.

**Table 3:** COPD-related factors associated with CAT score

Related factors	Univariate difference in CAT vs reference group (95% CI)	*p*-value	Proportion of CAT explained (R-squared value)	Multivariate difference in CAT vs reference group (95% CI)	*p*-value
% predicted FEV_1_	−0.10 (−0.13, −0.07)	<0.001	0.056	−0.004 (−0.03, 0.02)	0.763
Physical activity (IPAQ)					
Low	Reference		0.051	Reference	
Moderate	−3.27 (−4.87, −1.66)	<0.001		0.21 (−0.99, 1.40)	0.730
High	−4.91 (−6.71, −3.11)	<0.001		−0.34 (−1.71, 1.04)	0.632
Chronic cough/phlegm	4.89 (3.56, 6.21)	<0.001	0.067	3.11 (2.05, 4.18)	<0.001
Chronic wheeze	6.25 (4.84, 7.67)	<0.001	0.094	2.54 (1.37, 3.71)	<0.001
Exercise capacity (sit-to-stand repetition)	−0.54 (−0.64, −0.44)	<0.001	0.132	−0.11 (−0.20, −0.02)	0.014
Muscle strength (grip strength)	−0.13 (−0.19, 0.07)	<0.001	0.022	−0.04 (−0.09, 0.01)	0.088
MRC score					
1	Reference		0.461	Reference	
2	5.04 (2.56, 6.52)	<0.001		3.24 (1.73, 4.76)	<0.001
3	7.67 (6.24, 9.11)	<0.001		5.77 (4.24, 7.28)	<0.001
4	12.46 (11.00, 13.91)	<0.001		8.89 (7.19, 10.59)	<0.001
5	18.76 (17.14, 20.38)	<0.001		13.34 (11.28, 15.40)	<0.001
12-month exacerbations^a^					
None	Reference		0.098	Reference	
One	2.72 (1.00, 4.44)	0.002		0.79 (−0.55, 2.13)	0.247
Two or more	6.44 (5.02, 7.87)	<0.001		2.16 (0.96, 3.36)	<0.001
Respiratory hospitalization in the last 6 months	3.47 (1.11, 5.83)	0.004	0.010	0.45 (−1.34, 2.25)	0.621
Borg breathlessness					
Pre-sit/stand test	2.17 (1.83, 2.61)	<0.001	0.146	0.50 (0.11, 0.90)	0.012
Post-sit/stand test	2.15 (1.79, 2.50)	<0.001	0.163	0.51 (0.12, 0.89)	0.011

**Note:**

a Exacerbations were defined based on reported prescriptions for antibiotics or steroids for the participant’s lung condition over the previous 12 months.

**Abbreviations:** CAT, COPD Assessment Test; COPD, chronic obstructive pulmonary disease; FEV_1_, forced expiratory volume in 1 second; IPAQ, International Physical Activity Questionnaire; MRC, Medical Research Council.

**Table 4:** Psychosocial factors associated with CAT score

	Univariate CAT vs reference group (95% CI)	*p*-value	Proportion of HRQoL explained (R-squared)	Multivariate difference in CAT vs reference group (95% CI)	*p*-value
Lubben social network scale	−0.14 (−0.26, −0.02)	0.020	0.008	0.05 (−0.05, 0.14)	0.313
Anxiety (GAD-7)	0.88 (0.75, 1.02)	<0.001	0.215	−0.10 (−0.28, 0.09)	0.313
Depression (PHQ-9)	0.98 (0.88, 1.08)	<0.001	0.342	0.29 (0.11, 0.47)	0.002
Agoraphobic avoidance scale	6.01 (4.63, 7.38)	<0.001	0.097	1.32 (0.09, 2.54)	0.035
Nijmegen questionnaire	12.56 (11.29, 13.83)	<0.001	0.375	6.88 (5.33, 8.42)	<0.001
Brief IPQ	0.46 (0.42, 0.50)	<0.001	0.445	0.28 (0.23, 0.34)	<0.001

**Abbreviations:** CAT, COPD Assessment Test; COPD, chronic obstructive pulmonary disease; GAD-7, General Anxiety Disorder Assessment; HRQoL, health-related quality of life; IPQ, Illness Perception Questionnaire; PHQ-9, Patient Health questionnaire.

**Table 5:** Multivariate associations with CAT score – all significant variables (n=476)

	Difference in CAT vs reference group (95% CI)	*p*-value	Contribution to R-squared value	Proportion of R-squared explained, %	Ranking
MRC score at baseline			0.144	20.2	1
1	Reference				
2	2.97 (1.63, 4.31)	<0.001			
3	3.94 (2.55, 5.32)	<0.001			
4	4.46 (2.83, 6.09)	<0.001			
5	7.02 (5.04, 9.00)	<0.001			
Brief IPQ	0.16 (0.12, 0.21)	<0.001	0.142	19.8	2
Nijmegen score	4.72 (3.41, 6.02)	<0.001	0.125	17.5	3
Depression (PHQ-9)	0.19 (0.07, 0.31)	0.002	0.09	12.5	4
Exercise capacity (sit-to-stand repetition)	−0.13 (−0.22, −0.05)	0.001	0.038	5.3	5
Borg breathlessness	0.42 (0.07, 0.76)	0.017	0.037	5.1	6
12-month exacerbations^a^			0.029	4.0	7
None	Reference				
One	1.49 (0.29, 2.69)	0.015			
Two or more	1.73 (0.67, 2.80)	0.001			
Chronic wheeze	1.71 (0.64, 2.79)	0.002	0.024	3.4	8
IMD score quintile			0.024	3.3	9
1 (most deprived)	Reference				
2	−1.31 (−2.63, 0.004)	0.051			
3	−2.01 (−3.51, −0.52)	0.008			
4	−2.44 (−3.82, −1.05)	0.001			
5 (least deprived)	−2.81 (−4.28, −1.34)	<0.001			
Chronic cough/phlegm	1.90 (0.94, 2.87)	<0.001	0.021	2.9	10
Borg breathlessness pre-sit/stand test	−0.24 (−0.58, 0.11)	0.175	0.017	2.4	11
Agoraphobic avoidance	0.09 (−0.92, 1.10)	0.864	0.02	2.2	12
Smoking			0.01	1.2	13
Current smoker	Reference				
Ex-smoker	0.02 (−1.07, 1.12)	0.967			
Never smoked	−0.75 (−2.44, 0.95)	0.388			
Current working status			0.001	0.1	14
Employed	Reference				
Unemployed	0.68 (−0.80, 2.16)	0.368			
Retired	0.01 (−1.33, 1.34)	0.994			

**Note:**

a Exacerbations were defined based on reported prescriptions for antibiotics or steroids for the participant’s lung condition over the previous 12 months.

**Abbreviations:** CAT, COPD Assessment Test; COPD, chronic obstructive pulmonary disease; GAD-7, General Anxiety Disorder Assessment; IMD, Index of Multiple Deprivation; IPQ, Illness Perception Questionnaire; MRC, Medical Research Council; PHQ-9, Patient Health questionnaire.

**Table 6:** Multivariate associations with EQ-5D 5L – all significant variables (n=375)

	Difference in EQ-5D 5L vs reference group (95% CI)	*p*-value	Contribution to R-squared value	Proportion of R-squared explained, %	Ranking
Depression (PHQ-9)	−0.01 (−0.02, −0.01)	<0.001	0.139	23.1	1
MRC score at baseline			0.121	20.1	2
1	Reference				
2	−0.04 (−0.08, −0.004)	0.003			
3	−0.05 (−0.09, −0.01)	0.012			
4	−0.10 (−0.15, −0.06)	<0.001			
5	−0.16 (−0.22, −0.10)	<0.001			
Brief IPQ	−0.002 (−0.004, −0.001)	0.001	0.096	16.0	3
Anxiety (GAD-7)	−0.006 (−0.01, −0.001)	0.009	0.085	14.0	4
Dysfunctional breathing (Nijmegen scale)	−0.06 (−0.10, −0.01)	0.008	0.080	13.3	5
Exercise capacity (sit-to-stand repetition) at baseline	0.002 (−0.0004, 0.0005)	0.097	0.029	4.8	6
BMI			0.014	2.2	7
Normal	Reference				
Underweight	−0.07 (−0.16, 0.02)	0.151			
Overweight	−0.03 (−0.07, 0.004)	0.080			
Obese	−0.04 (−0.08, −0.01)	0.021			
Physical activity (IPAQ) score			0.013	2.2	8
Low	Reference				
Moderate	0.03 (0.002, 0.06)	0.032			
High	0.02 (−0.02, 0.06)	0.269			
Borg breathlessness pre-sit/stand test	0.002 (−0.007, 0.01)	0.670	0.013	2.1	9
Smoking			0.005	0.9	10
Current smoker	Reference				
Ex-smoker	0.01 (−0.02, 0.04)	0.521			
Never smoked	0.0003 (−0.05, 0.05)	0.988			
IMD score quintile			0.004	0.6	11
1 (most deprived)	Reference				
2	−0.01 (−0.06, 0.03)	0.543			
3	0.004 (−0.04, 0.05)	0.858			
4	−0.004 (−0.05, 0.04)	0.822			
5 (least deprived)	−0.01 (−0.06, 0.03)	0.626			
Chronic wheeze (wheeze ongoing for 3 consecutive months each year)	0.002 (−0.03, 0.03)	0.805	0.003	0.5	12
Current working status			0.002	0.3	13
Employed	Reference				
Unemployed	−0.01 (−0.05, 0.03)	0.602			
Retired	−0.01 (−0.06, −0.03)	0.523			

**Abbreviations:** BMI, body mass index; GAD-7, General Anxiety Disorder Assessment; IMD, Index of Multiple Deprivation; IPAQ, International Physical Activity Questionnaire; IPQ, Illness Perception Questionnaire; MRC, Medical Research Council; PHQ-9, Patient Health questionnaire; QoL, quality of life; EQ-5D 5L, 5 level EuroQoL questionnaire.
